# Emphysema Predicts Hospitalisation and Incident Airflow Obstruction among Older Smokers: A Prospective Cohort Study

**DOI:** 10.1371/journal.pone.0093221

**Published:** 2014-04-03

**Authors:** David A. McAllister, Firas S. Ahmed, John H. M. Austin, Claudia I. Henschke, Brad M. Keller, Adina Lemeshow, Anthony P. Reeves, Sonia Mesia-Vela, G. D. N. Pearson, Maria C. Shiau, Joseph E. Schwartz, David F. Yankelevitz, R. Graham Barr

**Affiliations:** 1 Centre for Population Health Sciences, University of Edinburgh, Edinburgh, United Kingdom; 2 Department of Medicine, College of Physicians and Surgeons, Columbia University, New York, New York, United States of America; 3 Department of Radiology, College of Physicians and Surgeons, Columbia University, New York, New York, United States of America; 4 Department of Radiology, Mount Sinai Medical Center, New York, New York, United States of America; 5 Department of Biomedical Engineering, University of Pennsylvania, Philadelphia, United States of America; 6 School of Electrical and Computer Engineering, College of Engineering, Cornell University, Ithaca, New York, United States of America; 7 Department of Radiology, New York University Langone Medical Center, New York, New York, United States of America; 8 Department of Epidemiology, Mailman School of Public Health, Columbia University, New York, New York, United States of America; Central Michigan University School of Medicine, United States of America

## Abstract

**Background:**

Emphysema on CT is common in older smokers. We hypothesised that emphysema on CT predicts acute episodes of care for chronic lower respiratory disease among older smokers.

**Materials and Methods:**

Participants in a lung cancer screening study age ≥60 years were recruited into a prospective cohort study in 2001–02. Two radiologists independently visually assessed the severity of emphysema as absent, mild, moderate or severe. Percent emphysema was defined as the proportion of voxels ≤ −910 Hounsfield Units. Participants completed a median of 5 visits over a median of 6 years of follow-up. The primary outcome was hospitalization, emergency room or urgent office visit for chronic lower respiratory disease. Spirometry was performed following ATS/ERS guidelines. Airflow obstruction was defined as FEV1/FVC ratio <0.70 and FEV1<80% predicted.

**Results:**

Of 521 participants, 4% had moderate or severe emphysema, which was associated with acute episodes of care (rate ratio 1.89; 95% CI: 1.01–3.52) adjusting for age, sex and race/ethnicity, as was percent emphysema, with similar associations for hospitalisation. Emphysema on visual assessment also predicted incident airflow obstruction (HR 5.14; 95% CI 2.19–21.1).

**Conclusion:**

Visually assessed emphysema and percent emphysema on CT predicted acute episodes of care for chronic lower respiratory disease, with the former predicting incident airflow obstruction among older smokers.

## Introduction

Chronic obstructive pulmonary disease (COPD) is the fourth leading cause of death worldwide [1] and chronic lower respiratory disease (CLRD), which comprises COPD, pulmonary emphysema, chronic bronchitis and asthma, recently overtook stroke as the third leading cause of death in the United States. [2].

Pulmonary emphysema is a common finding on computed tomography (CT) among older smokers. Approximately 25% of older smokers undergoing lung cancer screening,[3]–[5] 12% of patients investigated for pulmonary embolism, [6] and 4% of patients undergoing cardiac CT scans have detectable emphysema on CT. [7] Given that lung cancer CT screening has recently been found to improve lung cancer survival among smokers, [8] pulmonologists are likely to encounter emphysema on CT in increasing numbers of patients.

Emphysema assessed quantitatively or visually on CT scans is correlated with airflow obstruction on spirometry [9]–[14] but also occurs in the absence of spirometrically defined COPD, for example, in a recent large multi-centre study of patients with COPD, radiologists identified mild to moderate emphysema in 8.5% of the control group, which comprised smokers with normal spirometry. [15].

Emphysema on CT predicts all-cause mortality and death from COPD and lung cancer [16]–[18]. Emphysema also predicts decline in pulmonary function in both COPD [19] and in male older current and former heavy smokers [20], but did not independently predict exacerbations in a large prospective study of mostly severe COPD. [21] However, whether quantitative and qualitative measures of emphysema predict acute episodes of care for lower respiratory diseases among older smokers is unknown.

We therefore examined whether visual and quantitative measures of emphysema on CT independently predicted acute episodes of care for CLRD and decline in lung function in a prospective cohort study of older current or former smokers undergoing CT screening for lung cancer.

## Methods

We enrolled all 557 participants at one site of a lung cancer CT screening study in 2001–2002. [22] Inclusion criteria were ≥10 pack-years of cigarette smoking, age 60 years and older, willingness to undergo screening for lung cancer, and no cancer history except non-melanoma skin cancer. Thirty-six (6%) participants were missing percent emphysema measures due to irretrievable scans; therefore 521 participants were included.

The baseline and annual follow-up visits included a CT scan, spirometry, and assessment of acute episodes of CLRD and potential confounders. Annual follow-up visits continued through July 01, 2009.

The Institutional Review Board of Columbia University and the Western Institutional Review Board approved all study activities and all participants provided written informed consent.

### Emphysema on CT Scan

All participants underwent low-dose, non-contrast, full-lung CT scanning on a single Siemens 4-slice multidetector scanner in 2001–02 (140 kVp, 43 mAs, 10-mm thickness, B50f reconstruction kernel, contiguous slices from the thoracic inlet to the adrenals) in a protocol consistent with the National Lung Cancer Screening trial. [23].

#### Qualitative visual emphysema assessment

Two board-certified academic chest radiologists read all sections of all scans prior to reviewing participant information and without percent emphysema scores; 61% of scans were re-read a third time. Emphysema was graded as none, mild, moderate or severe. The intra-observer and inter-observer agreement by weighted Kappa score were 0.91 and 0.72 respectively. Agreement in distinguishing moderate or severe emphysema from no emphysema was excellent (Kappa = 0.95) whereas agreement in distinguishing mild emphysema from none was modest (Kappa = 0.65). Where readers disagreed, the majority opinion was taken except in 27 discordant cases with two reads only, for which the earlier read was used.

#### Quantitative percent emphysema

The percentage of emphysema-like lung, hereafter referred to as percent emphysema, was defined as the proportion of full lung volume with attenuation ≤ –910 Hounsfield Units (HU). [13] To correct for interscan variation in air attenuation, we modified the base threshold using the attenuation of tracheal air.

### Acute Episodes of Care for CLRD

#### Definition

Acute episodes of care for CLRD were identified via questionnaire at annual follow-up visits. Participants were asked the number of times that they had been hospitalized or had an urgent clinic or emergency room visit for COPD, bronchitis, emphysema or asthma. Acute episodes were defined as the sum of urgent office visits, emergency room visits and hospitalizations. [24].

#### Electronic medical records

Since self-reported acute episodes relied upon completeness of follow-up, we also identified these from electronic medical records for all 214 participants whose primary doctor was affiliated with Columbia University Medical Center or who resided in zip codes adjacent to the hospital. Acute episodes were defined as hospitalizations, emergency room visits, and clinic attendances assigned a primary or secondary diagnostic code of acute exacerbation of COPD or asthma (ICD-9 CM 491.21, 491.22, 493.02, 493.21, 493.22, or 493.92). Hospitalizations for any respiratory disease (ICD-9 CM 460 to 519) were also identified. Repeat episodes within 13 days were treated as single events. [25].

### Spirometry

Spirometry was performed in 2001–02 and annually thereafter according to American Thoracic Society (ATS) guidelines [26] using the EasyOne Diagnostic spirometer (ndd Medical Technologies, Chelmsford, MA), which we have previously validated. [27] Measurements were reviewed by a physician and spirometry quality was defined using 2005 ATS/European Respiratory Society (ERS) recommendations. [28] Airflow obstruction was defined as a FEV1/forced vital capacity (FVC) ratio <0.70 and FEV1<80% predicted. [29] NHANES prediction equations were used.

### Covariate Data

Information on age, sex, race/ethnicity, educational attainment, clinical diagnoses, medication and healthcare insurance status was recorded by interviewer-administered questionnaire at baseline and follow-up visits. Suspected chronic lower respiratory disease at baseline was defined as self-reported COPD, asthma, emphysema or chronic bronchitis or prescriptions of inhaled beta-agonists, anticholinergics or corticosteroids, or methylxanthines. Body mass index (BMI) was calculated as weight (kg) divided by height squared (m^2^). Smoking status was verified by urinary cotinine levels, ascertained using enzyme-linked immunosorbent assay (Orasure Technologies, Inc., Bethlehem, PA).

### Statistical Analysis

Baseline characteristics were compared across quartiles of percent emphysema. Initial stratum-specific rate ratios (RR) were calculated as the number of acute episodes of care divided by the years from the baseline visit to last follow-up. Multivariate RRs for all acute episodes and for hospitalizations were estimated using generalized linear models with a Poisson distribution. Rates were weighted for follow-up time and standard errors were corrected for overdispersion. [30] Initial models adjusted for the confounders of age, sex, and race/ethnicity and full models additionally adjusted for pack years, cotinine at time of CT, private healthcare insurance (as a marker of socioeconomic status) and suspected chronic lower respiratory disease. We separately report associations additionally adjusted for pulmonary function (FEV1 and the FEV1/FVC ratio) as in the majority of clinical settings spirometry is unlikely to have been performed prior to CT lung cancer screening on all patients.

To ensure that these findings are not solely the result of a small number of participants experiencing multiple recurrent events, we performed secondary analyses for time-to-first-event using hazard ratios (HR) in Cox proportional hazards models.

The relationship of emphysema at baseline to change in FEV1 was assessed with linear mixed models adjusting for age, sex, race/ethnicity, height, height^2^, weight, weight^2^, smoking status and pack years at baseline, and urinary cotinine levels. These models accommodate variable follow-up times assuming missing-at-random, in other words, that the missing data is not related to emphysema conditional on the information in the model. In Cox-proportional hazard models we also estimated the instantaneous risk of testing positive for airflow obstruction on follow-up spirometry where it was not present at baseline.

Missing covariate data were uncommon. Six respondents aged <65 years with missing healthcare insurance data were assumed to be uninsured and, in 4 participants without urinary cotinine, the sample median was used.

Analyses were performed using SAS version 9.2 (SAS Institute, Cary, NC) and R version 2.14.2 (R Foundation for Statistical Computing, Vienna, Austria).

## Results

The mean age of the 521 participants was 67 years at baseline, 51% were male and 42% were current smokers. Eighteen percent had mild and 3% had moderate or severe emphysema assessed visually on CT scan. Thirty-six percent had airflow obstruction on spirometry.

Participants with higher percent emphysema had lower lung function, greater dyspnea and more self-reported COPD than participants with less percent emphysema (Table 1). Moderate or severe emphysema on visual assessment increased across quartiles of percent emphysema. Visual emphysema assessment and percent emphysema were moderately correlated (r = 0.10, P = 0.02) but were both associated with the FEV1/FVC ratio (r = −0.28; P<0.001 and r = −0.31, P<0.001 respectively).

**Table 1:** Baseline characteristics by CT percent emphysema quartiles.

	Q1	Q2	Q3	Q4
Number of participants	130	131	129	131
Median percent emphysema, %	9	20	31	47
Age, mean (SD), years	66 (6)	66 (4)	68 (5)	68 (6)
Gender – male, n (%)	55 (42)	77 (59)	74 (57)	60 (46)
Race/Ethnicity, n (%)				
Caucasian	87 (67)	93 (71)	94 (73)	111 (85)
African-American	17 (13)	13 (10)	10 (8)	5 (4)
Hispanic	18 (14)	13 (10)	8 (6)	7 (5)
Chinese	9 (7)	12 (9)	17 (13)	8 (6)
Height, mean (SD), cm	168 (9)	170 (10)	170 (10)	169 (10)
Weight, mean (SD), kg	76 (15)	80 (16)	78 (15)	72 (15)
BMI, mean (SD), kg/m^2^	27 (5)	27 (5)	27 (5)	25 (4)
BMI <25 kg/m^2^, n (%)	51 (39)	46 (35)	48 (37)	73 (56)
BMI 25 to 30 kg/m^2^, n (%)	49 (38)	50 (38)	48 (37)	45 (34)
BMI >30 kg/m^2^, n (%)	30 (23)	35 (27)	34 (26)	13 (10)
Educational Attainment, n (%)				
Graduate Degree	31 (24)	25 (19)	40 (31)	41 (31)
Batchelor Degree	38 (29)	37 (28)	36 (28)	34 (26)
High School/some College	48 (37)	58 (44)	44 (34)	50 (38)
No High School Diploma	13 (10)	10 (8)	9 (7)	7 (5)
Healthcare Insurance, n (%)				
Private	74 (57)	64 (49)	71 (55)	77 (59)
Medicare	31 (24)	47 (36)	31 (24)	41 (31)
Other Government	12 (9)	10 (8)	12 (9)	9 (7)
None	12 (9)	8 (6)	15 (12)	4 (3)
Diagnosis of, n (%)				
Asthma	13 (10)	18 (14)	6 (5)	20 (15)
Emphysema or chronic bronchitis	7 (5)	17 (13)	17 (13)	25 (19)
Acute episode of care for CLRD, n (%)	9 (7)	8(6)	4(3)	16(13)
Respiratory symptoms, n (%)				
MRC Chronic Bronchitis	29 (22)	28 (21)	23 (18)	26 (20)
MRC Grade 3 Dyspnea or worse	12 (9)	8 (6)	13 (10)	14 (11)
Smoke Exposure				
Current Smoker, n (%)	73 (56)	60 (46)	35 (27)	47 (36)
Pack Years, median (IQR)	46 (36–67)	48 (34–63)	44 (28–65)	41 (27–59)
Cotinine, median (IQR), ng/ml	967 (25–2047)	90 (20–1583)	32 (11–291)	62 (19–1216)
Spirometry				
FEV1 percent predicted, mean (SD), %	80 (18)	82 (18)	81 (21)	76 (23)
FVC percent predicted, mean (SD), %	85 (18)	89 (18)	90 (18)	88 (19)
FEV1/FVC ratio, mean (SD),%	72 (8)	70 (9)	68 (10)	65 (12)
FEV1/FVC ratio <0.70and FEV1<80% predicted, n (%)	33 (25)	38 (29)	52 (40)	63 (48)
Visual emphysema assessment, n (%)				
None	103 (79)	106 (81)	99 (77)	94 (72)
Mild	26 (20)	17 (13)	26 (20)	26 (20)
Moderate or Severe	1 (1)	8 (6)	4 (3)	10 (8)

FEV1 - forced expiratory volume in one second, FVC - forced vital capacity, CLRD – chronic lower respiratory disease (includes any of asthma, COPD, chronic bronchitis and emphysema).

The 521 participants completed a median of 5 visits over a median of 6 years of follow-up. Twenty-four died without attending follow-up and 384 (77%) completed at least one follow-up visit, with 324 (65% overall and 84% of those with one or more follow-up visits) completing a visit 5 or more years after baseline. Percent emphysema and baseline lung function were similar in those with and without follow-up.

### Acute Episodes of Care for CLRD

Ninety participants reported 287 episodes of urgent care, with 29 reporting 51 hospitalizations, over 1,897 person-years of follow-up for mean rates of 15.1 acute episodes and 2.7 hospitalizations per hundred person-years. Of the 90 participants, 78 had self-reported COPD, asthma, emphysema or chronic bronchitis or prescriptions of inhaled beta-agonists, anticholinergic or corticosteroids, or methylxanthines. Of these 20 had airflow obstruction at baseline and 15 subsequently had airflow obstruction on follow-up spirometry.

Adjusting for age, sex, race/ethnicity, cotinine, healthcare insurance, height and history of known COPD, compared to participants without airflow obstruction, rates for acute episodes of care were higher for participants with FEV1 percent predicted 50 to 79% (RR 1.60; 95% CI: 1.03–2.47; P = 0.03) and for participants with FEV1 percent predicted less than 50% (RR 3.38; 95% CI: 1.91–6.00; P<0.001). Adjusting for the same covariates, higher rates were also found for hospitalisation for participants with FEV1 percent predicted 50 to 79% (RR 3.82; 95% CI: 2.18–6.69; P<0.001) and for participants with FEV1 percent predicted less than 50% (RR 4.52; 95% CI: 2.08–9.87; P<0.001).

### Visual Emphysema Assessment

#### Acute episodes of care for CLRD

Moderate to severe emphysema on visual assessment was associated with acute episodes of care (RR 1.89; 95% CI: 1.01–3.52; P = 0.046) and hospitalisation (RR 2.90; 95% CI: 1.56–5.42; P<0.001) adjusting for age, sex and race/ethnicity (Table 2). After additional adjustment for pack years smoking, cotinine at time of CT, private healthcare insurance and suspected chronic lower respiratory disease the associations for both acute episodes of care (RR 1.58; 95% CI: 0.83–2.99; P = 0.16) and hospitalisation (RR 2.17; 95% CI: 1.11–4.24; P = 0.02) were attenuated, although the latter remained statistically significant. The associations were strongly attenuated by the inclusion of pulmonary function at baseline (RR 1.09; 95% CI 0.58–2.05; P = 0.78 and RR 1.61; 95% CI: 0.83–3.14; P = 0.16 respectively). Mild emphysema on visual assessment was not associated with acute episodes of care or hospitalisation.

**Table 2:** Episodes of chronic lower respiratory disease by category of emphysema visually.

	Noemphysema	Mildemphysema	PValue	Moderate tosevereemphysema	PValue
Number of participants	402	95		23	
***Acute episodes of care***					
Number of episodes	234	28		25	
Total follow-up time (years)	1488	334		75	
Number of episodes per ten person years	1.57	0.84		3.33	
Rate Ratio					
Model 1	1	0.56 (0.31–1.01)	0.06	1.89 (1.01–3.52)	0.046
Model 2	1	0.61 (0.34–1.10)	0.10	1.58 (0.83–2.99)	0.16
***Hospitalization***					
Number of hospitalizations	37	7		7	
Total follow-up time (years)Total follow-up time (years)	1488	334		75	
Number of hospitalizations per ten person years	0.02	0.02		0.09	
Rate Ratio					
Model 1	1	0.82 (0.44–1.54)	0.55	2.90 (1.56–5.42)	<0.001
Model 2	1	1.11 (0.59–2.07)	0.75	2.17 (1.11–4.24)	0.02
***Airflow obstruction on follow-up spirometry***					
Number of participants who developed airflow obstruction over follow-up	56	46		7	
Number of participants without airflow obstruction at baseline	293	15		9	
Model 1	1	1.85 (1.04–3.28)	0.04	7.29 (3.25–16.4)	<0.001
Model 2	1	1.44 (0.79–2.62)	0.23	5.14 (2.19–12.1)	<0.001

Model 1 adjusted for age, sex and race/ethnicity.

Model 2 additionally adjusted for pack years smoking, cotinine at time of CT, private healthcare insurance and suspected chronic lower respiratory disease.

One person who had percent emphysema measured did not have emphysema assessed visually.

#### Decline in lung function

When pulmonary function was modeled as a continuous variable neither mild nor moderate to severe emphysema predicted decline in FEV1 (2 ml per year; 95% CI −21 to 24 and 1 ml per year; 95% CI −13 to 11 respectively, P = 0.98). Findings were similar for decline in the FEV1/FVC ratio, FEV1 percent predicted and proportional change in FEV1 (P = 0.98, P = 0.95 and P = 0.20 respectively).

However, among participants without airflow obstruction at baseline (Table 2), moderate to severe emphysema was associated with increased risk of airflow obstruction on follow-up spirometry after adjusting for age, sex and ethnicity (HR 7.29; 95% CI 3.25–16.4;P<0.001) and after pack years smoking, cotinine at time of CT, private healthcare insurance and suspected chronic lower respiratory disease (HR 5.14; 95% CI 2.19–21.1; P<0.001). Weaker associations in the same direction were observed for mild emphysema.

### Percent Emphysema

#### Acute episodes of care for CLRD

**Figure 1:**
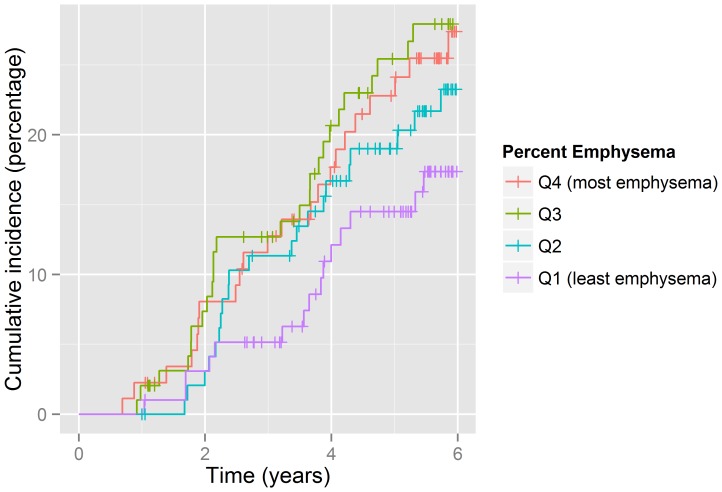
Cumulative incidence plot of time to first episode by percent emphysema.

Percent emphysema also predicted acute episodes of care (Table 3, Figure 1). Participants in the highest quartile of percent emphysema had approximately twice the rate as those in the lowest quartile (RR 2.40; 95% CI: 1.37–4.21) and more than three times the rate of hospitalization for CLRD (RR 3.50; 95% CI: 1.69–7.24). Associations were attenuated by approximately 10% after adjusting for height, FEV1 and FEV1/FVC ratio but the association persisted.

**Table 3:** Episodes of chronic lower respiratory disease by category of percent emphysema.

	Q1	Q2	Q3	Q4	One SD Change in CTPercent Emphysema	P Value
Number of participants	130	131	129	131		
***Acute episodes of care***						
Number of episodes	49	88	72	78		
Total follow-up time (years)	463	477	515	442		
Number of episodes per ten person years	1.06	1.84	1.40	1.76		
Rate Ratio						
Model 1	1	2.04	1.64	2.40	1.29 (1.08–1.55)	0.006
Model 2	1	1.81	1.58	2.49	1.33 (1.11–1.60)	0.002
***Number of participants with ≥1 episode of care***	16	22	27	25		
Hazard Ratio for first episode						
Model 1	1	1.69	1.96	2.15	1.32 (1.07–1.63)	0.008
Model 2	1	1.63	1.89	2.30	1.35 (1.09–1.68)	0.005
***Hospitalizations***						
Number of hospitalizations	7	19	13	12		
Total follow-up time (years)	463	477	515	442		
Number of hospitalizations per ten person years	0.15	0.40	0.25	0.27		
Rate Ratio						
Model 1	1	3.36	1.78	3.50	1.44 (1.15–1.80)	0.002
Model 2	1	3.03	1.99	3.54	1.47 (1.19–1.81)	<0.001

Model 1 adjusted for age, sex and race/ethnicity.

Model 2 additionally adjusted for pack years smoking, cotinine at time of CT, private healthcare insurance and suspected chronic lower respiratory disease.

The association for percent emphysema was not modified by including emphysema assessed visually in the model (RR 1.33; 95% CI: 1.11–1.60; P = 0.002).

Similar results were obtained when time to first episode was modeled in cox-proportional hazard models.

#### Decline in lung function

Percent emphysema was not associated with airflow obstruction on follow-up spirometry (HR 1.05 per one SD; 0.84–1.33; P = 0.66). Nor did percent emphysema predict differences in the decline of FEV1 (−1 ml/year per SD increment in percent emphysema, 95%CI −6 to 4, P = 0.62) nor incident airflow obstruction.

### Additional Analyses

Among 214 local participants for whom electronic medical records were available, there were 28 episodes of care for CLRD and 21 admissions for any respiratory disease over 1,540 person-years. Percent emphysema predicted episodes of care (multivariate RR 1.45; 95% CI: 1.04–2.03; P = 0.03) and respiratory admissions (multivariate RR 1.62; 95% CI: 1.08–2.44; P = 0.02) adjusting for age, sex, race/ethnicity and cotinine at time of CT scan.

## Discussion

In older smokers undergoing lung cancer screening, patients with moderate or severe emphysema on CT scan had a two-fold increased rate of hospitalization for CLRD after adjustment for smoking and other potential confounders over a median of six years of follow-up. Quantitative percent emphysema was associated with an increased rate for acute episodes of care and hospitalization for CLRD.

The present study adds to the literature by demonstrating strong relationships of both qualitatively and quantitatively defined emphysema on CT scan to hospitalisation for CLRD in a prospective cohort study of older smokers with and without COPD. No prior studies of which we have aware have reported this association for radiologist-defined pulmonary emphysema, which is the clinically relevant measure of pulmonary emphysema and which could assist in risk-stratification of smokers.

Qualitatively and quantitatively defined emphysema were only weakly correlated with each other, although both were similarly associated with the FEV1/FVC ratio at baseline, and percent emphysema continued to predict acute episodes of care and hospitalisations for CLRD after adjusting for emphysema on visual assessment. As such qualitative and quantitative emphysema measures may provide complementary clinical information.

The present longitudinal results for percent emphysema are consistent with and build upon those of a prior large cross-sectional study of patients with COPD [31] but are at variance with those of a large longitudinal study of patients with COPD. [21] The latter study, however, enrolled patients predominantly with severe COPD and 47% of enrolled participants reported an exacerbation in the year prior to enrolment, compared to 17% in the cross-sectional study and 8% in the present study. The present findings for both percent emphysema and visually assessed emphysema remained significant after adjustment for prior exacerbations. These divergent results suggest that emphysema on CT scan may predict clinically important events among patients with milder and no COPD but do not predict eventsindependently of frequent exacerbations among patient with severe COPD.

Two recent large studies demonstrated that emphysema predicted a small but statistically significant accelerated decline in the FEV1 [19], [20] (13 ml and 27 ml per year respectively). We also found that compared to participants without emphysema on CT, those participants with moderate to severe emphysema had a five-fold increased risk of new airflow obstruction on follow-up spirometry, with similar but weaker associations for mild emphysema. This finding persisted after adjusting for smoking and additional potential confounders. However, caution is needed in interpreting our finding in view of the small numbers with moderate to severe emphysema, and because we did not find that emphysema predicted decline in lung function in our primary analysis.

There has been recent interest in strategies for case-finding to reduce harms and costs associated with hospitalisation. [32] Our findings and those of Hoesein et al [20] suggest that consideration should be given to COPD case-finding among older smokers with emphysema identified on CT lung cancer screening, since not only was airflow obstruction common both at baseline and subsequently, but subsequent hospitalisation for CLRD was also commoner among this group. Studies in this group of patients investigating the efficacy and cost-effectiveness of spirometry for preventing hospitalisation are needed.

### Limitations

Follow-up in this eight-year study of smokers was incomplete, leading to a likely underestimation of the rates of acute episodes of care, hospitalization and decline in lung function. However, follow-up did not differ by category of percent emphysema, and percent emphysema predicted acute episodes and hospitalization in a subset where these outcomes were identified via electronic medical records; therefore, the results are unlikely to be the result of differential follow-up. Radiologists did not follow a published protocol such as that of the National Emphysema Treatment Trial (NETT) to grade emphysema. [33] Although this may have reduced precision, reproducibility for moderate or severe emphysema was high and this approach likely increased clinical applicability since the interpretations followed standard academic clinical practice. Moreover, using an identical method this group of radiologists found that mild, moderate and severe emphysema predicted lung cancer and mortality from COPD in older smokers undergoing CT lung cancer screening. [16] An additional limitation was that reproducibility for mild emphysema was not high. Therefore, it is unclear if mild emphysema failed to predict clinical events because it is truly of no clinical significance or because of the imprecision of the measure. The results for percent emphysema make the latter more likely, in our opinion.

Although both qualitatively and quantitatively defined emphysema were statistically significantly associated with hospitalisation for CLRD, only the latter was associated for all acute episodes of care for CLRD. However, the observed associations were similar across hospitalisation and all acute episodes of care for both measures, and as such we suggest that that the most likely explanation for the lack of statistical significance for moderate to severe emphysema on visual assessment is the lower precision of “all episodes” as an outcome measure compared to “hospitalisation”.

Lung function was measured without a bronchodilator, which reduced precision. However, the mean decline in the FEV1 was similar to that reported for pre-bronchodilator spirometry in the UPLIFT trial in COPD. [34].

In conclusion, both radiologist-determined and quantitatively defined pulmonary emphysema on CT scan independently predicted hospitalizations for CLRD with the former also predicting incident airflow limitation in a general sample of older smokers.
